# In Vitro Metabolism and In Vivo Pharmacokinetics Profiles of Hydroxy-α-Sanshool

**DOI:** 10.3390/toxics12020100

**Published:** 2024-01-24

**Authors:** Jie Meng, Die Qian, Ruo-Lan Li, Wei Peng, Li Ai

**Affiliations:** 1 State Key Laboratory of Southwestern Chinese Medicine Resources, School of Pharmacy, Chengdu University of Traditional Chinese Medicine, Chengdu 611137, China; mengjie2009@126.com (J.M.); qdcdtcm9925@163.com (D.Q.); lee69205@163.com (R.-L.L.); 2 State Key Laboratory of Southwestern Chinese Medicine Resources, School of Ethnic Medicine, Chengdu University of Traditional Chinese Medicine, Chengdu 611137, China; 3 Sichuan Chinese Medicinal Decoction Pieces Co., Ltd., Chengdu 611732, China

**Keywords:** hydroxy-α-sanshool, liver microsomes, metabolism, pharmacokinetics profile

## Abstract

Hydroxy-α-sanshool (HAS) is the predominant active compound in *Zanthoxylum bungeanum* Maxim (ZBM). Our present work was aimed to explore the in vitro metabolism characteristics, and in vivo pharmacokinetic (PK) profile of HAS. Plasma (human), liver microsomes, and hepatocytes (human, monkey, dog, mouse, and rat) were collected for HAS metabolism studies in vitro and HAS elimination rates in liver microsomes and hepatocytes of different species were investigated. In addition, five recombinant human CYP enzymes were used to identify CYP isoforms of HAS. Finally, the PK properties of HAS in rats in vivo were studied by oral administration (p.o.). The results showed that HAS stably metabolized in human and rat liver microsomes and human hepatocytes, and the binding of HAS to human plasma proteins was nonspecific; HAS has strong inhibitory effects on CYP2C9 and CYP2D6 of human liver microsomes. In addition, in vivo PK study, HAS is rapidly absorbed in rats after oral administration. In conclusion, the in vivo and in vitro metabolic studies of HAS in this study provide data support for its further development and application, and the metabolic profiles of different species can be used as a reference for its safety evaluation.

## 1. Introduction

Hydroxy-α-sanshool (HAS) is the main active component in the fruits of *Zanthoxylum bungeanum* Maxim (ZBM) that has been the subject of its research [1,2,3]. As a natural and well-known spice in China, ZBM has attracted increasing attention. HAS is the most important flavor constituent corresponding to its pungent flavor [3]. More notably, it has been intensively studied due to its rich pharmacological activities such as hypolipidemic, hypoglycemic, anti-inflammatory, neuroprotective, antioxidant, and other effects [4,5,6,7]. The structure of HAS has been studied more clearly and belongs to the fatty chain amide alkaloids. It consists of a total of 16 carbon atoms (C_16_H_25_NO_6_) and is also systematically named as (2E, 6Z, 8E, 10E)-N-isobutyl-2, 6, 8, 10-dodecatetraenamide [2,3]. Our previous study demonstrated that HAS has an antidiabetic effect on experimental diabetic mice, and the underlying mechanism may be related to modulation of Akt/GSK3β/GS signaling [6]. In addition, we also found that HAS has an anti-obesity effect by inducing white fat browning, increasing energy expenditure and improving the metabolic profile of the body, which leads to weight loss; more importantly, it was found that the potential drug target for this process could be the membrane receptor of TRPV1 [8].

Pharmacokinetics (PK) mainly investigates the absorption (A), distribution (D), metabolism (M), and excretion (E) of drugs in body, which is used to assess efficacy, safety and toxicity of drugs. At present, pharmacokinetic studies that focus only on HAS are not comprehensive [9]. Therefore, it is necessary to study the potential drug-forming activity of HAS separately. In the past, studies have been involved in studying the PK of the main active ingredients in ZBM including HAS, hydroxy-β-sanshool (HBS), and hydroxy-γ-sanshool (HRS) using both subcutaneous and intravenous injections, and the results showed that after subcutaneous administration, the drug was rapidly absorbed and widely distributed in the plasma, and it was noted that the absolute bioavailability of HAS was high [10]. In addition, preclinical studies of drugs need to involve studies related to the stability of drug metabolism in vivo or in vitro, such as metabolic stability, protein binding, and bioavailability [11]. Furthermore, analyzing the metabolism of the drug in different species in vitro is helpful in predicting the role of the drug in human organism metabolism and safety evaluation [12]. However, the PK properties and metabolic stability of HAS in different species have not been investigated.

In this study, our aim was to investigate the distribution and metabolic stability of HAS in different species in vitro, as well as PK in vivo, in order to predict the suitability of HAS for use in clinical trials. The study of HAS’s drug-forming properties was mainly reflected in the following aspects, including the binding rate of HAS to human plasma proteins; the effects of HAS on metabolizing enzymes (cytochrome P450, CYP); the metabolic stability of HAS in liver microsomes, hepatocytes in different species, and finally, the study of the PK of HAS’s action in SD rats were performed.

## 2. Results

### 2.1. In Vitro Plasma Protein Binding (PPB)

As listed in Table 1, the in vitro PPB results of HAS in human plasma. Ketoconazole was used as the reference compound for testing PPB. And we found that HAS was very strong non-specific binding in human plasma to obtain significant protein binding data. The recovery of HAS in human species was 6.76%. At 6 h, the remaining was greater than 99.92%.

### 2.2. CYP Inhibition in Human Liver Microsomes

**Figure 1:**
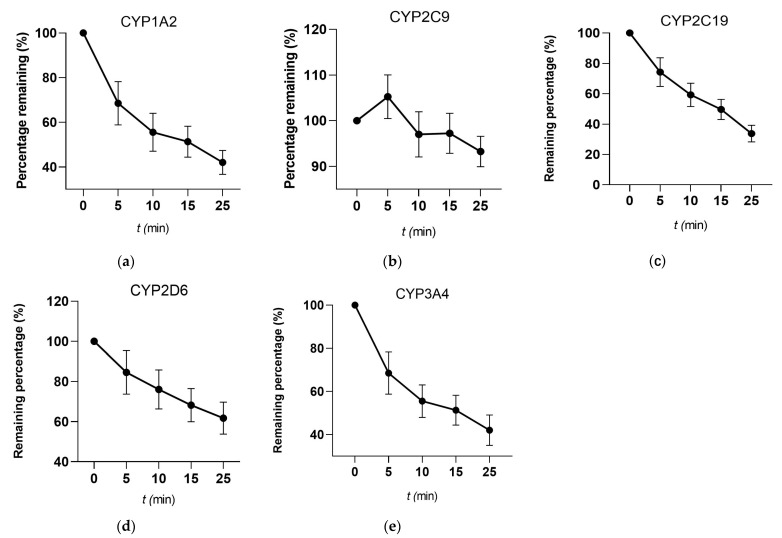
Line graph of remaining percentages of HAS in different recombinant human CYP enzymes with NAPDH. (**a**) CYP1A2; (**b**) CYP2C9; (**c**) CYP2C19; (**d**) CYP2D6; and (**e**) CYP3A4. Data were expressed as Mean ± SD (n = 3).

CYP Isoform Identification of HAS with 5 Recombinant Human CYP Enzymes. The results showed in Figure 1 and Table 2. The percentage remaining of the test compounds in different recombinant human CYP enzymes containing NADPH was determined at 0, 5, 10, 15, and 25 min. With the extension of time, the remaining percentage gradually decreases. At 25 min, the percentage remaining after the action of HAS on phenacetin (CYP1A2), s-mephenytoin (CYP2C19), and midazolam (CYP3A4) was less than 50%, suggesting that HAS has a weak inhibitory effect on these three CYP enzymes. And they were shown to have a shorter half-life. However, HAS had a strong inhibitory effect on diclofenac (CYP2C9) and dextromethorphan (CYP2D6), with remaining percentages of 93.24% and 61.77%, respectively, and CYP2C9 inhibitor had a long half-life of 185.67 min. These findings suggest that HAS may be the potent inhibitors of CYP2C9 and CYP2D6.

### 2.3. Metabolic Stability in Liver Microsomes

**Figure 2:**
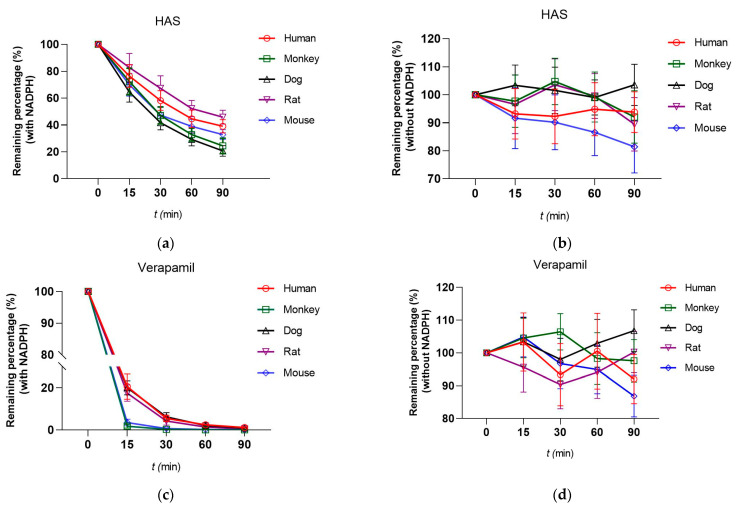
The metabolic stability curve with NADPH (**a**,**c**) and without NADPH (**b**,**d**) of HAS and Verapamil in liver microsomes of humans, monkeys, dogs, rats, and mice. Data were expressed as Mean ± SD (n = 3).

Liver microsomes (human, monkey, dog, rat, and mouse) were used to determine the metabolic stability of HAS. The results of metabolic stability of HAS and Verapamil (VRP) in different species of liver microsomes were shown in Table 3 and Figure 2. Compared to Verapamil, HAS had a longer half-life for metabolic stability in liver microsomes of different species and a lower intrinsic clearance in vitro, suggesting that HAS was more stable. The residual amounts of HAS in the five species (human, monkey, dog, rat, and mouse) were 38.99%, 24.46%, 20.69%, 45.77%, and 32.82%, respectively, after the 60-minute-reaction under the condition of accompanied NADPH. Without NADPH involved in the reaction, there was more HAS remaining, both greater than 50%. Although HAS was metabolized in monkey, dog, and mouse liver microsomes with shorter elimination half-life (T_1/2_) and more intrinsic clearance (CL_int_), the T_1/2_ in humans and rat were 42.92 min, 51.38 min, and CL_int_ were 40.50 and 48.34 mL/min/kg, respectively, which were between 30–70%, indicating that HAS was metabolized more stably in the liver microsomes of humans and rats.

### 2.4. Metabolic Stability in Hepatocytes

**Figure 3:**
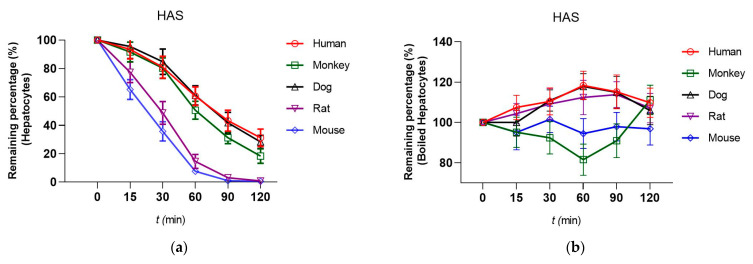

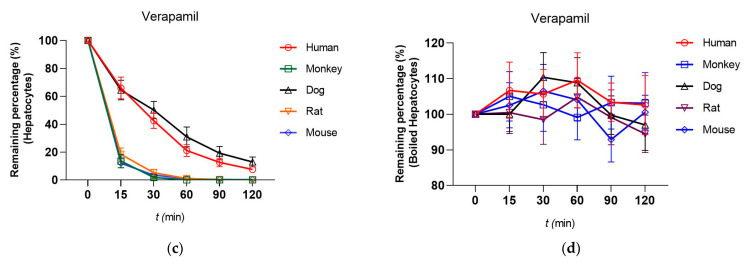
The metabolic stability curve of HAS and Verapamil in hepatocytes and boiled hepatocytes of humans, monkeys, dogs, rats, and mice. (**a,c**) in hepatocytes; (**b,d**) in boiled hepatocytes. Data were expressed as Mean ± SD (n = 3).

The metabolic stability of HAS as well as the positive control compound (Verapamil, VRP) in hepatocytes of five different species was researched. As shown in Table 4 and Figure 3, based on the half-life T_1/2_ values, the T_1/2_ of HAS in human and dog hepatocytes were 69.59 min, 63.74 min, and the Cl_int_ values were 50.67, 149.60 mL/min/kg, respectively. Moreover, after incubation for 120 min, the residual amounts of HAS in humans, monkeys, dogs, rats, and mice hepatocytes were 31.24%, 18.35%, 28.30%, 0.84%, and 0.51%, respectively. Positive control compounds showed higher in vitro clearance.

### 2.5. PK Profiles of HAS in Rats

**Figure 4:**
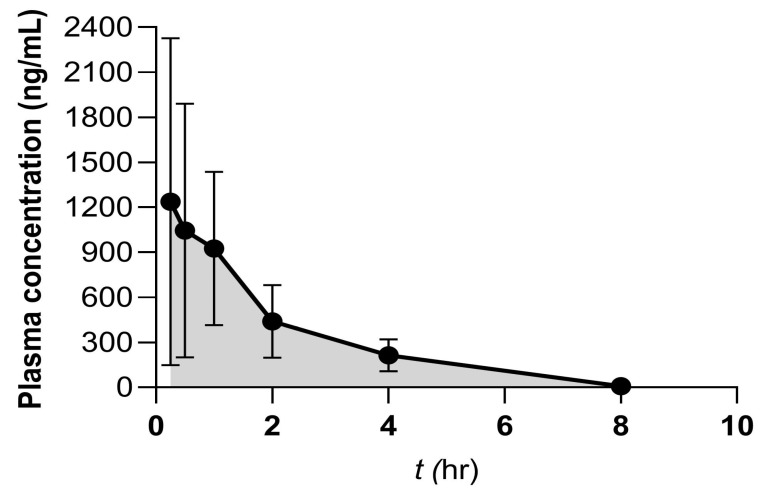
The plasma concentration-time profiles of HAS at 10 mg/kg oral administration in rats. Data were expressed as Mean ± SD (n = 3).

Subsequently, PK properties of HAS in rats were investigated in vivo after oral administration (10 mg/kg, p.o.), and the PK profile was shown in Table 5 and Figure 4. SD rat plasma was collected and quantified analyzed by HPLC-MS/MS for PK studies. The plasma concentration-time curves of HAS in rats showed that the mean value of maximum blood concentration (C_max_) after oral administration was 1253 ± 1075 ng/mL. In addition, the areas under the plasma concentration–time curve extrapolated to the last time point (AUC_last_) after oral administration was 2714 ± 1637 h × ng/mL. HAS was administered orally to SD rats and the T_1/2_ value for drug elimination in vivo was determined to be 1.02 ± 0.16 h. Furthermore, the absorption rate constant (TvKa) value was 1.816 ± 2.261 (1/h), and the time of maximum concentration (T_max_) was 0.75 ± 0.43 h, suggesting HAS was rapidly absorbed into action after oral administration. Collectively, the results revealed that HAS has good PK properties, moderate clearance, suitable in vivo T_1/2_, and effective in vivo exposure levels, indicating that HAS is appropriate as a drug candidate for further studies.

## 3. Discussion

Currently, accumulating lends support to the view that traditional Chinese medicines (TCMs) and their monomers are important resources for finding various candidate drugs [13,14]. Pharmacokinetic studies of TCMs are of great significance in revealing the active substances and mechanisms of TCMs, designing and optimizing the route of administration of TCM, promoting the new drugs development, improving the dosage form, and controlling the quality of TCM [15]. It is well known that drug metabolism reflects the results of drug–drug interactions and has the highest incidence of interactions among the four phases of PK [16]. Considering the fact that HAS serves as the main active ingredient of ZBM, which is traditionally used for both medicinal and dietary purposes, and that there has been a great deal of research shown that HAS has a wealth of pharmacological effects and plays a potentially advantageous role in the treatment of diabetes, obesity, fatty liver, and other diseases [4,5,6,7]. Therefore, the metabolic stability of this monomer in different species in vivo and in vitro has been investigated, including plasma protein binding, liver microsomes, hepatocytes metabolic stability, and PK studies, to provide reference and evaluation indexes for the new drug development of HAS.

Drug metabolism cannot be achieved without the involvement of metabolizing enzymes, especially the CYP450 enzyme family, which is the most common [17]. CYP450 enzymes are found in various human tissues and organs, with the liver and small intestine contributing the most to the overall metabolism and elimination of drugs [18]. In addition, CYP450 enzymes distributed in liver tissues, CYP3A4, CYP2E,1 and CYP2C9 are the most important CYP enzymes in body [19]. In our present work, inhibitory effects of HAS on CYP enzyme (CYP1A2, CYP2C19, CYP3A4, CYP2C9, and CYP2D6) have been investigated. These are five the recombinant human CYP enzymes commonly used to study drug metabolism, and the results showed a more significant inhibition of CYP2C9 and CYP2D6 by HAS. The study reflected clinically significant drug–drug interactions, preventing adverse effects of drug therapy as well as predicts therapeutic outcomes [20].

Plasma protein binding (PPB), as the name suggests, is the binding of plasma proteins after the drug enters the body, which helps to understand the basic rule of drug action [21]. The binding rate of a drug to plasma proteins is one of the important parameters of pharmacokinetics, which affects the disposition of the drug in the body (including drugs’ distribution, metabolism, excretion), thus affecting the efficacy and safety of the drug. After the drug enters the bloodstream, part of the drug will bind with plasma proteins to form bound drug, while the free drug will be distributed to various tissues with the flow of blood and finally reach the target organ to exert therapeutic effect [22,23]. In this experiment, Ketoconazole was used as a control, which showed a high binding rate of 98.53% to human plasma proteins and an unbound rate of 1.47%, whereas HAS showed a strong non-specific binding capacity to human plasma proteins. HAS compound may competitively bind to plasma proteins when co-administered with other drugs, resulting in an increase in unbound drug, which may result in enhanced efficacy or adverse effects. In addition, it should be pointed out that the shortcomings of this experiment did not involve the difference in the binding of HAS to plasma proteins of other species. Perhaps, the drug-forming property of HAS can be further evaluated by follow-up experiments.

Metabolic stability is also a metric that needs to be examined in drug design, indicating the susceptibility of a compound to biotransformation, and the results are represented by the in vitro T_1/2_ and CL_int_ [11]. Effective delivery of intact drugs to functional targets for action, rather than being hydrolyzed midway, which means that enhanced metabolic stability of the drug is essential for its enhanced efficacy [24]. Zhu et al. assessed the inhibitory activity of Ko143 analogs against ABCG2 by investigating the binding mode, cytotoxicity, and metabolic stability with ABCG2, and found that the amide-modified compounds were more metabolically stable [25]. Thus, it is a good research idea to help develop new formulations by improving the stability of drug metabolism. In addition, a comparison of drug metabolism in different species is important to elucidate drug clearance based on drug safety considerations.

Investigation of the differences of metabolic stability of HAS in different animals would be beneficial for predicting the safety and metabolic profile of HAS in the human body [12]. Monkey, dog, rat, and mouse are the commonly used animal model for preclinical drug research. Therefore, in this study, metabolic stability of HAS in liver microsomes and hepatocytes (human, monkey, dog, rat, and mouse) were investigated using HPLC-MS/MS method, and the main influences on its kinetic properties were evaluated in terms of T_1/2_, CL_int_ and remaining fraction (%).The metabolic stability of HAS in liver microsomes of different species was determined by using Verapamil as a control drug, and the results showed that HAS was stable in human liver microsomes, with a T_1/2_ of 42.92 min and a CL_int_ of 40.50 mL/min/kg, while it was more stable in rat liver microsomes, with a T_1/2_ of 51.38 min and a CL_int_ of 48.34 mL/min/kg. In contrast, comparison with other species revealed that HAS had higher drug clearance in dog and mouse liver microsomes, reaching 130.99 and 165.07 mL/min/kg, respectively, suggesting metabolic instability. In addition, the results of metabolic stability of HAS in human hepatocytes and in human liver microsomes turned out to be similar, with a T_1/2_ of 69.59 min and a CL_int_ of 50.67 mL/min/kg, whereas it was metabolized more rapidly in monkey, dog and mouse hepatocytes.

As we all know, liver microsomes and hepatocytes are commonly used as models for the study of drug metabolism in vitro, which have slight differences [26]. Liver microsomes are mainly derived from hepatocyte endoplasmic reticulum enzymes, and drugs are directly exposed to metabolite enzymes, whereas hepatocytes possess an intact cellular structure and compounds need to penetrate the cell membrane to enter the cell for metabolism [11]. This explains the fact that drug metabolism in some species results in more remaining hepatocytes than liver microsomes. Furthermore, the addition of NADPH is required to initiate the reaction when liver particles are used for CYP450 enzyme studies [27]. The present results revealed HAS remaining (%) liver microsomes of different species was different between conditions with and without NADPH.

Further, in SD rat PK study, it was administered by oral dosing, which is different from the previous study of ZBM pharmacokinetics [10]. Generally, PK properties are reflected based on PK parameters such as T_1/2_, T_max_, C_max_, and AUC values. After oral administration, the absorption of HAS into the rat body was rapid, and it took only 0.75 h to reach the maximum drug concentration of 1253 ng/mL. The elimination half-life can directly reflect the rate of drug elimination from the body [11], and the mean T_1/2_ of HAS was 1.02 h. It can be seen that HAS was absorbed quickly in rats, but was not maintained in the body long enough to be eliminated more quickly. In addition, the results showed that HAS compound fully exposed in rats with a good degree of absorption. Therefore, it is important to prolong the maintenance time of HAS efficacy in vivo, improve oral bioavailability, and develop a rational dosing regimen to improve the pharmacological efficacy of HAS.

## 4. Materials and Methods

### 4.1. Materials

Hydroxy-α-sanshool was purchased from Chengdu Bencao Tianyun Biotechnology Co., Ltd. (Chengdu, China), and the purity was over than 98% for HPLC detection (Chengdu, China). Ketoconazole (Lot# SLBB5223V). NADPH, phenacetin (CYP1A2), diclofenac (CYP2C9), amitriptyline, and testosterone were acquired from Sigma-Aldrich (Shanghai, China). S-mephenytoin (CYP2C19), dextromethorphan (CYP2D6), and midazolam (CYP3A4) were procured from CYPEX agency (Beijing, China). Human liver microsomes (male and female) were purchased from the Corning (Shanghai, China); liver microsomes of monkey (Cynomolgus), dog (Beagle), rat (SD), and mouse (CD-1) were provided from the Bioreclamation IVT (Shanghai, China). Human hepatocytes, monkey hepatocytes (Cynomilgus), dog hepatocytes (Beagle), rat hepatocytes (SD), and mouse hepatocytes (CD-1) were procured from Bioreclamation IVT (Shanghai, China). William’s E medium, human recombinant insulin, glutaMAX, and HEPES were procured from Life technologies (Shanghai, China). Isotonic percoll was purchased from General Electric (Boston, MA, USA); Fetal bovine serum (FBS) was acquired from Corning (Shanghai, China); Dexamethasone was provided from Pharmaron. Inc. (Beijing, China).

### 4.2. Ethical Statement

All experimental protocols related animals obeyed the Care and Use of Laboratory Animals published by the US NIH (NIH Publication, revised 2011) and approved by the Animal Care and Use Committee of Chengdu University of Traditional Chinese Medicine (Approval No. 2020-27).

### 4.3. Methods

#### 4.3.1. Plasma Protein Binding (PPB)

PPB assays were carried out using the HTD 96-well equilibrium DIALYZERTM (Htdialysis, Gales Ferry, CT, USA). First, the basic solution (solution A) consisted of Na_2_HPO_4_ (14.2 mg/mL) and NaCl (8.77 mg/mL) in water, and the acidic solution (solution B) was consisted with NaH_2_PO_4_ (12.0 mg/mL) and NaCl (8.77 mg/mL) in deionized water. Then, the (solution A) was titrated with the (solution B) to pH 7.4 to prepare the dialysis buffer (solution C). The quench solution (solution D) consisted of Alprazolam (100 nM) and Ketoprofen (2 μM) in acetonitrile.

Total 3 μL of testing plasm samples (dissolved in DMSO, 200 μM) was mixed with 597 μL of plasma (the final concentration was 1 μM). The dialysis membranes were pre-soaked with water (60 min), then with 20% ethanol (20 min), and with dialysis buffer (20 min). Both the plasm samples and the dialysis buffer are 100 μL. Then, the dialysis plate was sealed and incubated at 37 °C with 5% CO_2_ at 100 rpm for 6 h. After 6 hours’ incubation, 50 μL of samples of the dialysis buffer and plasma samples were collected and re-added to a 96-well plate. Then, 50 μL of blank plasma was added to buffer samples, and 50 μL PBS was added to plasma samples. Subsequently, 300 μL quench solution was added to precipitate protein. Then, the samples were collected and centrifuged at 3220× *g* (30 min at 4 °C), and then the supernatant was collected for HPLC-MS/MS analysis (the detail HPLC-MS/MS conditions were described in the supplementary files including Table S1–S5).

The Fu% was calculated as follows:

(1) $$\%Fu=\frac{{Peak\;Area\;Ratio}_{buffer\;chamber}}{{Peak\;Area\;Ratio}_{plasma\;chamber}}\times 100$$

(2) %Bound=100−%Fu

(3) $$\%Recovery=\frac{{Peak\;Area\;Ratio}_{buffer\;chamber}+{Peak\;Area\;Ratio}_{plasma\;chamber}}{{Peak\;Area\;Ratio}_{total\;sample}}\times 100$$

where:

Peak Area Ratio _buffer chamber_ means the conc for free fraction;

Peak Area Ratio _plasma chamber_ means the conc for both free and bound fraction;

Peak Area Ratio _total sample_ means the conc for starting sample before incubation.

#### 4.3.2. CYP Inhibition in HLMs

Five recombinant CYP enzymes were performed to determine the HAS’s metabolic stability, including CYP1A2, CYP2C9, CYP2C19, CYP2D6 and CYP3A4. A master solution containing recombinant human CYP enzymes (100 pmoL/mL) and phosphate buffer (100 mM) was prepared, and pre-warmed at 37 °C for 15 min. 2.5 μL of testing compound or control compound working solution was added to each pre-warmed master solution. The final concentration of control compound and test compound in the reaction system was 2 μM. Then, 27 µL of the CYP/compound mixture was transferred from the incubation plate to the 0 min “Quenching plates” containing 300 µL of cold quench solution (Alprazolam (100 nM) and Ketoprofen (2 μM) in acetonitrile) and 3 µL 10 mM NADPH. Then, the reaction was initiated with the addition of 22 μL of 10 mM NADPH solution to the incubation plate. At 5, 10, 15, and 25 min, the incubation mixture was mixed on a whirly mixer for 10 s and 30 µL was transferred at each time point to wells in a separate “Quenching plate” containing 300 µL of cold quench solution, which was sealed with a lid and kept on ice. Samples were centrifuged at 3220× *g* for 40 min to precipitate protein. And the supernatant was collected for HPLC-MS/MS analysis (the detail HPLC-MS/MS conditions were described in the supplementary files including Table S1–S5). The in vitro T_1/2_ was calculated using the following Formula (4):

(4) $$in\;vitro\;T_{1/2}=-\frac{0.693}{k}$$

where:

The slope value, *k*, was determined by linear regression of the natural logarithm of the remaining percentage of the parent drug vs. incubation time curve.

Conversion of the in vitro T_1/2_ (min) into the in vitro CL_int_ in µL/min/pmoL P450 was done using the following Equation (5):

(5) $$in\;vitro\;{CL}_{int}=\left(\frac{0.693}{T_{1/2}}\right)\times (\frac{volume\;of\;incubation\;(µL)}{amount\;of\;CYP450\;(pmoL)})$$

#### 4.3.3. Metabolic Stability in Liver Microsomes

The metabolic stability of HAS in human (Lot#: 38295), monkey (Lot#: 0041001CNC), dog (Lot#: QRZ), rat (Lot#: 0112002), and mouse (Lot#: 1029001) liver microsomes was determined. The specific experimental methodology is as follows: two separate experiments were performed, one with the addition of 20 mg/mL liver microsomes and 10 mM NADPH 40 μL, and the other with 20 mg/mL liver microsomes and water 40 μL without NADPH.

The reaction started by adding 4 μL testing sample (100 μM, final concentration was 1 μM) at 37 °C. Then, aliquots of 50 µL were collected from the reaction solution at 0, 15, 30, 45, and 60 min. The reaction was stopped by adding cold quench solution [Alprazolam (200 nM) and Ketoprofen (2 μM) in acetonitrile]. Samples were centrifuged at 3220× *g* for 40 min, and the supernatant was collected for HPLC-MS/MS analysis (the detail HPLC-MS/MS conditions were described in the supplementary files including Tables S1–S5). Conversion of the in vitro T_1/2_ (min) into the in vitro CL_int_, in µL/min/mg protein was done using the following Equation (6):

(6) $$in\;vitro\;{CL}_{int}=\left(\frac{0.693}{T_{1/2}}\right)\times (\frac{volume\;of\;incubation\;(µL)}{amount\;of\;proteins\;(mg)})$$

Conversion of the in vitro T_1/2_ (min) into the scale-up unbound intrinsic clearance (Scale-up CL_int_, in mL/min/kg) was done using the following Equation (7):

(7) $$Scale\;up\;{CL}_{int}=\left(\frac{0.693}{T_{1/2}}\right)\times \left(\frac{volume\;of\;incubation\;\left(µL\right)}{amount\;of\;proteins\;\left(mg\right)}\right)\times Scaling\;Factor$$

where:

(Scaling Factor: human-1254.2; monkey-1500.0; dog-2492.8; rat-1792.0; and mouse-4400.0)

#### 4.3.4. Metabolic Stability in Hepatocytes

The hepatocyte metabolic stability of HAS in human (Lot#: RHZ), monkey (Lot#: GSYG), dog (Lot#: UXH), rat (Lot#: WHU), and mouse (Lot#: IEP) hepatocytes was determined. Hepatocytes of different species were normally cultured in a 5% CO_2_, 37 °C cell culture incubator to a certain concentration and inoculated in 96-well plates. Total 2 μL of the 100 μM testing samples were added into respective wells of the 96-well non-coated plate to start the reaction. Remove well contents in 25 μL aliquots at time points of 0, 15, 30, 60, 90, and 120 min. The aliquots were mixed with 6 volumes (150 μL) of cold quench solution (Alprazolam (100 nM), labetalol (200 nM), caffeine (200 nM), and Ketoprofen (2 μM) in acetonitrile) to stop reaction. Samples were centrifuged for 20 min at 3220× *g*. the supernatant was collected for HPLC-MS/MS analysis (the detail HPLC-MS/MS conditions were described in the supplementary files including Tables S1–S5).

Conversion of the in vitro T_1/2_ (min) into the scale-up intrinsic clearance (Scaled-up CL_int_, in mL/min/kg) was done using the following equation (mean of duplicate determinations):

(8) $$Scale\;up\;{CL}_{int}=\left(\frac{kV}{N}\right)\times Scaling\;Factor$$

where:

K = rate constant (-slope value); V = incubation volume (0.2 mL); N = number of hepatocytes per well (0.1 × 10^6^ cells). Scaling Factor: human-2544.3, monkey-3600.0, dog-6880.0, rat-4680.0, and mouse-11812.5.

#### 4.3.5. PK Experiments in Rats and Analysis

PK assay of HAS was performed in male Sprague Dawley (SD) rats by oral administration (n = 3). Each rat weighed 200 ± 20 g and was acquired from Sibeifu (Beijing, China) BioTech. (Beijing, China) for pharmacokinetic evaluation. Before the start of the study, the rats were fed with water and maintenance feed to acclimate for a week. The rats were administered with HAS (10 mg/kg) orally and blood samples were collected at 0.25, 0.5, 1, 2, 4, 8, and 24 h after oral administration. The collected blood volume at each time point was 0.25 mL. In PK study, rat plasma was mainly selected, which can reflect the distribution of drugs. Therefore, rat plasma samples were separated from blood samples by centrifugation at 12,000× *g* for 15 min and stored frozen in a −80 °C refrigerator prior to use.

After the time-concentration data were obtained from the HPLC-MS/MS analysis (the detail HPLC-MS/MS conditions were described in the supplementary files including Tables S1–S5), they were imported into the WinNonlin Professional software (version 8.3, Pharsight, Sunnyvale, CA, USA), and a non-compartmental model was constructed to calculate PK parameters such as area under the curve (AUC) and T_1/2_. After oral administration, the T_1/2_, T_max_, C_max_, AUC_last_, AUC_Inf_, AUC__%Extrap_obs_, MRT_Inf_obs_, AUC_last_/D, and bioavailability (F %) were calculated. The final results are expressed as mean ± standard deviation.

## 5. Conclusions

In summary, according to the in vitro experimental results, the binding between HAS and plasma proteins exhibits strong non-specific binding; HAS has strong inhibitory effects on CYP2C9 and CYP2D6 of human liver microsomes, indicating that when used in combination with other drugs, it may enhance its efficacy or produce adverse reactions; the metabolism of HAS is relatively stable in human and rat liver microsomes and human hepatocytes. In addition, in vivo pharmacokinetic study, HAS is rapidly absorbed in rats, and combined with its rich pharmacological activity, it is believed that HAS has the potential to be used as a new drug for development.

## Figures and Tables

**Table 1:** Protein binding of HAS in human plasma.

Compound	Bound (%)	Recovery (%)	Remaining at 6 h (%)	Fu (%)
Ketoconazole	98.53 ± 4.76	92.61 ± 7.48	110.91 ± 6.04	1.47 ± 0.68
HAS	NA	6.76 ± 1.87	99.92 ± 8.75	NA

NA: Not applicable, HAS was very strong non-specific binding in human plasma to obtain significant protein binding data. Data were expressed as Mean ± standard deviation (SD) (n = 3). **Bound values**: >90%: highly binding; 50–90%: moderate binding; <50%: low binding. Fu (%), unbound fraction (%).

**Table 2:** Metabolic stability of HAS in different recombinant human CYP enzymes.

Compound	CYP Types	Concentration	T_1/2_ (min)	CL_int_ (μL/min/pmoL CYP450)
HAS	CYP1A2	2 μM	12.76 ± 2.48	0.540 ± 0.15
HAS	CYP2C9	2 μM	185.67 ± 19.54	0.037 ± 0.01
HAS	CYP2C19	2 μM	16.32 ± 5.39	0.420 ± 0.12
HAS	CYP2D6	2 μM	36.65 ± 7.25	0.190 ± 0.07
HAS	CYP3A4	2 μM	15.66 ± 3.13	0.440 ± 0.16

Data were expressed as Mean ± SD (n = 3).

**Table 3:** T_1/2_ and CL_int_ of HAS in liver microsomes.

Species	Human		Monkey		Dog	
	VRP	HAS	VRP	HAS	VRP	HAS
T_1/2_ (min)	9.53 ± 1.87	42.92 ± 5.53	2.60 ± 0.46	28.81 ± 8.02	8.18 ± 2.67	26.38 ± 3.31
CL_int_ (μL/min/mg protein)	145.48 ± 7.04	32.29 ± 4.91	532.65 ± 82.79	48.10 ± 6.27	169.44 ± 43.07	52.55 ± 8.85
CL_int_ (mL/min/kg)	182.46 ± 23.18	40.50 ± 8.02	798.97 ± 159.48	72.15 ± 5.03	422.38 ± 98.49	130.99 ± 7.69
**Species**	**Rat**		**Mouse**			
	**VRP**	**HAS**	**VRP**	**HAS**		
T_1/2_ (min)	7.36 ± 1.22	51.38 ± 7.49	3.12 ± 0.87	36.94 ± 4.23		
CL_int_ (μL/min/mg protein)	188.23 ± 14.64	26.97 ± 4.27	444.22 ± 84.25	37.52 ± 5.04		
CL_int_ (mL/min/kg)	337.31 ± 79.62	48.34 ± 6.59	1954.57 ± 379.01	165.07 ± 6.38		

**CL_int_ values**: >70% means high clearance; 30–70% means moderate clearance; <30% means low clearance. VRP, Verapamil. Data were expressed as Mean ± SD (n = 3).

**Table 4:** T_1/2_ and CL_int_ of HAS in hepatocytes of different species.

Species	Human		Monkey		Dog	
	VRP	HAS	VRP	HAS	VRP	HAS
T_1/2_ (min)	32.43 ± 9.43	69.59 ± 11.46	5.19 ± 1.75	47.78 ± 8.39	42.01 ± 7.42	63.74 ± 7.05
CL_int_ (μL/min/10^6^ cells)	42.73 ± 4.97	19.92 ± 3.28	266.98 ± 31.61	29.01 ± 4.22	32.99 ± 6.44	21.74 ± 4.29
CL_int_ (mL/min/kg)	108.72 ± 8.93	50.67 ± 9.37	961.13 ± 143.29	104.43 ± 13.16	227.00 ± 32.94	149.6 ± 21.05
**Species**	**Rat**		**Mouse**			
	**VRP**	**HAS**	**VRP**	**HAS**		
T_1/2_ (min)	9.38 ± 2.15	17.52 ± 4.13	9.62 ± 1.91	15.83 ± 3.28		
CL_int_ (μL/min/10^6^ cells)	147.79 ± 22.19	79.10 ± 6.48	144.15 ± 8.02	87.53 ± 6.29		
CL_int_ (mL/min/kg)	691.68 ± 98.43	370.20 ± 65.66	1702.72 ± 269.47	1033.92 ± 147.27		

**CL_int_ values**: >70% means high clearance; 30–70% means moderate clearance; <30% means low clearance. VRP, Verapamil. Data were expressed as Mean ± SD (n = 3).

**Table 5:** The PK parameters of HAS administered orally in rats.

Dose	T_1/2_ (h)	T_max_ (h)	TvKa (1/h)	C_max_ (ng/mL)	AUC_last_ (h×ng/mL)	AUC_lnf_ (h×ng/mL)	AUC__%Extrap_obs_ (%)	MRT_Inf_obs_ (h)	AUC_last_/D (h×ng/mL)	F (%)
10 mg/kg (p.o.)	1.02 ± 0.16	0.75 ± 0.43	1.816 ± 2.261	1253 ± 1075	2714 ± 1637	2726 ± 1642	0.517 ± 0.347	1.92 ± 0.17	271 ± 164	NA

Tv: typical value; Ka: absorption rate constant; AUC: areas under the plasma concentration -time curve; MRT: mean residence time. Data were expressed as Mean ± SD (n = 3).

## Data Availability

The raw data supporting the conclusions of this article will be made available by the authors on request.

## Supplementary Materials

The following supporting information can be downloaded at: https://www.mdpi.com/article/10.3390/toxics12020100/s1, Methods: HPLC-MS/MS conditions used in this paper, Table S1: Plasma Protein Binding-The chromatographic conditions for analysis, Table S2: CYP Inhibition in HLMs-The chromatographic conditions for analysis, Table S3: Metabolic Stability in Liver Microsomes-The chromatographic conditions for analysis, Table S4: Metabolic Stability in Hepatocytes-The chromatographic conditions for analysis, Table S5: PK Experiments in Rats and Analysis-The chromatographic conditions for analysis.
