# Supplementary material for: In Vitro Metabolism and In Vivo Pharmacokinetics Profiles of Hydroxy-α-Sanshool
Source: Toxics. 2024 Jan 24;12(2):100. doi: 10.3390/toxics12020100 (PMC10891682; doi:10.3390/toxics12020100)
Supplement: Supplementary file 1 [file toxics-12-00100-s001.zip › toxics-2821650-supplementary.pdf]

HPLC-MS/MS conditions used in this paper

1. Plasma Protein Binding

The essential information regarding the LC/MS conditions of analysis.

1. 1. Chromatographic conditions

LC system: Shimadzu

MS analysis: Triple QuadTM 5500 instrument from AB Inc (Canada) with an ESI interface

Column temperature: 40 °C

Injection volume: 2 μL

Column: XSelect Hss T3 2.5μ (2.1 × 50 mm) Column XP coupled with preguard column

Elution rate: 0.6 mL/min

Mobile phase: 0.1% formic acid in water (A) and 0.1% formic acid in acetonitrile (B)

Table S1 The chromatographic conditions for analysis.

Time (min)	0	0.3	0.8	1.5	1.6	2.0
% B	5	5	100	100	5	5

1.2. MS parameters

Ion source: Turbo spray

Ionization model: ESI

Scan type：MRM

Collision gas: 10L/min

Curtain gas: 30L/min

Nebulize gas: 60L/min

Auxiliary gas: 60 L/min

Temperature: 550°C

Ionspray voltage: + 5500 v (positive MRM)

2. CYP Inhibition in HLMs

The essential information regarding the LC/MS conditions of analysis.

2.1. Chromatographic conditions

LC system: Shimadzu

MS analysis: Triple QuadTM 5500 instrument/ Triple QuadTM 6500+ instrument from AB Inc (Canada) with an ESI interface

Column temperature: 40 °C

Injection volume: 1 μL

Column: XSelect Hss T3 2.5 μm (2.1×50 mm) Column XP

Elution rate: 1.0 mL/min

Mobile phase: 0.1% formic acid in water (A) and 0.1% formic acid in acetonitrile (B)

Table S2. The chromatographic conditions for analysis.

Time (min)	0.00	0.30	0.60	0.90	0.91	1.20
%B	5	5	100	100	5	5

2.2. MS parameters

Ion source: Turbo spray

Ionization model: ESI

Scan type：MRM

Collision gas: 10L/min

Curtain gas: 30L/min

Nebulize gas: 60L/min

Auxiliary gas: 60 L/min

Temperature: 550°C

Ionspray voltage: + 5500 v (positive MRM)

3. Metabolic Stability in Liver Microsomes

The essential information regarding the LC/MS conditions of analysis.

3.1. Chromatographic conditions

LC system: Shimadzu

MS analysis: API 4000 instrument from AB Inc (Canada) with an ESI interface

Column temperature: 40 °C

Injection volume: 3 μL

Column: Waters XSelect HSS T3 C18, 2.5μm, 2.1 x 50mm

Elution rate: 1.0 mL/min

Mobile phase: 0.1% formic acid in water (A) and 0.1% formic acid in acetonitrile (B)

Table S3 The chromatographic conditions for analysis.

Time (min)	0.00	0.10	0.50	0.80	0.81	1.00
%B	3	3	100	100	3	3

3.2. MS parameters

Ion source: Turbo spray

Ionization model: ESI

Scan type：MRM

Collision gas: 10L/min

Curtain gas: 30L/min

Nebulize gas: 50L/min

Auxiliary gas: 50 L/min

Temperature: 550°C

Ionspray voltage: +5500 v (positive MRM) / -4500 v (negative MRM)

4. Metabolic Stability in Hepatocytes

The essential information regarding the LC/MS conditions of analysis.

4.1. Chromatographic conditions

LC system: Shimadzu

MS analysis: API 4000 instrument from AB Inc (Canada) with an ESI interface

Column temperature: 40 °C

Injection volume: 3 μL

Column: Waters XSelect HSS T3 C18, 2.5μm, 2.1 x 50mm

Elution rate: 1.0 mL/min

Mobile phase: 0.1% formic acid in water (A) and 0.1% formic acid in acetonitrile (B)

Table S4 The chromatographic conditions for analysis.

Time (min)	0.00	0.30	0.50	1.20	1.30	1.40
%B	5	5	100	100	5	5

4.2. MS parameters

Ion source: Turbo spray

Ionization model: ESI

Scan type：MRM

Collision gas: 10L/min

Curtain gas: 30L/min

Nebulize gas: 50L/min

Auxiliary gas: 50 L/min

Temperature: 550°C

Ionspray voltage: +5500 v (positive MRM) / -4500 v (negative MRM)

5. PK Experiments in Rats and Analysis

The essential information regarding the LC/MS conditions of analysis.

5.1. Chromatographic conditions

LC system: Shimadzu

MS: LCMS-8060 instrument (Serial No. 011105400182 AE)

Column temperature: 40 °C

Injection volume: 5 μL

Column: Raptor Biphenyl C18, 2.7μm, 2.1 x 50mm

Elution rate: 0.6mL/min

Mobile phase: 0.1% formic acid in water (A) and 0.1% formic acid in acetonitrile (B)

Table S5. The chromatographic conditions for analysis.

Time (min)	0.20	1.50	1.90	1.91	2.30
%B	5	95	95	5	5

5.2. MS parameters

Ion source: Turbo spray

Ionization model: ESI

Scan type：MRM

Collision gas: 10L/min

Curtain gas: 30L/min

Nebulize gas: 50L/min

Auxiliary gas: 50 L/min

Temperature: 550°C

Ionspray voltage: +5500 v (positive MRM) / -4500 v (negative MRM)
